# ﻿A new species of *Pseudopoda* (Araneae, Sparassidae) from China, with the description of different and distinctive internal ducts of the female vulva

**DOI:** 10.3897/zookeys.1159.97463

**Published:** 2023-05-02

**Authors:** Li-jun Gong, Meng-yun Zeng, Yang Zhong, Hui-liang Yu

**Affiliations:** 1 School of Nuclear Technology and Chemistry & Biology, Hubei University of Science and Technology, Xianning 437100, Hubei, China; 2 Hubei Key Laboratory of Radiation Chemistry and Functional Materials, Hubei University of Science and Technology, Xianning 437100, Hubei, China; 3 Administrative Commission of Jiugongshan National Nature Reserve of Hubei Xianning, Xianning, 437100, Hubei, China; 4 Shennongjia National Park Administration, Shennongjia 442421, Hubei, China; 5 Hubei Province Key Laboratory of Conservation Biology for Shennongjia Golden Monkey, Shennongjia 442421, Hubei, China

**Keywords:** DNA barcoding, Hubei, huntsman spiders, morphology, taxonomy

## Abstract

One new species of the genus *Pseudopoda* Jäger, 2000, *Pseudopodadeformis* Gong & Zhong, **sp. nov.** (♂, ♀), is described and documented with digital images from Shennongjia Forestry District, Hubei Province, China, based on morphology and DNA barcodes. This new species is separated from other *Pseudopoda* species by the unique type of internal ducts of the female vulva that are curved longitudinally, forming a narrow triangle or trapezoidal shape. In addition, DNA barcodes for this species are provided.

## ﻿Introduction

*Pseudopoda* Jäger, 2000 is currently the largest genus in the family Sparassidae Bertkau, 1872. It comprises 251 species, of which 152 are recorded from China, representing 60.6% of the global species (WSC 2023). The genus has been recorded in areas from South Asia (49 species in Nepal, India, Bhutan, and Pakistan), East Asia (154 species in China and Japan) and Southeast Asia (50 species in Myanmar, Thailand, Laos, and Vietnam) (WSC 2023).

While examining specimens recently collected from Shennongjia Forestry District of Hubei Province, central China, we found some huntsman spiders. The spiders described in this paper were identified as a new species based on comparison with other *Pseudopoda* species. The male palp of this new species has a slender embolus, and the female vulva has unique internal ducts. We used DNA barcodes of the species to match the sexes and for future use in identification.

## ﻿Material and methods

Specimens were examined and measured with an Olympus SZX7 stereomicroscope. Positions of tegular appendages are given according to clock positions, based on the left palp in ventral view. Male and female copulatory organs were examined and illustrated after dissection from the spider bodies; vulvae were cleared in a warm 10% potassium hydroxide (KOH) solution. All photographs were captured with a KUY NICE industrial digital camera (20.0 megapixels) mounted on an Olympus CX43 dissecting microscope and assembled using Helicon Focus 3.10.3 image stacking software. Photographic images were then edited using Adobe Photoshop CC 2018. All measurements were obtained using an Olympus SZX7 stereomicroscope and are given in millimetres (mm).

Leg measurements are shown as: total length (femur, patella, tibia, metatarsus, tarsus). Number of macrosetae is listed for each segment in the following order: prolateral, dorsal, retrolateral, ventral; in femora and patellae ventral spines are absent and thus the fourth article is omitted in the setation formula (Gong and Zhong 2020).

Abbreviations used in the text and figures are given below: **ALE** = anterior lateral eye, **AME** = anterior median eye, **AW** = anterior width of carapace, **C** = conductor, **CO** = copulatory opening, **CH** = clypeus height, **dRTA** = dorsal branch of RTA, **E** = embolus, **FD** = fertilisation duct, **Fe** = femur, **LL** = lateral lobes, **Mt** = metatarsus, **OL** = opisthosoma length, **OW** = opisthosoma width, **Pa** = patella, **PI** = posterior incision of LL, **PL** = carapace length, **PLE** = posterior lateral eyes, **PME** = posterior median eyes, **Pp** = palp or palpus, **PW** = carapace width, **RTA** = retrolateral tibial apophysis, **Sp** = spermophor, **T** = tegulum, **Ta** = tarsus, **Ti** = tibia. I, II, III, IV—legs I to IV, **vRTA** = ventral branch of RTA, **HUST** = School of Nuclear Technology and Chemistry and Biology, Hubei University of Science and Technology, Xianning, Hubei, China.

To obtain DNA barcodes, one mitochondrial gene (mitochondrial cytochrome oxidase subunit I [COI]) and one nuclear gene (Internal Transcribed Spacer 2 [ITS2]) were amplified and sequenced for four specimens. Primers (Folmer et al. 1994), PCR conditions and other information (e.g., extraction, amplification and sequencing procedures) are the same as in Zhang et al. (2021). The accession numbers are provided in Table 1. For phylogenetic inference, we used the dataset (COI + ITS2) from Cao et al. (2016) and added the new sequences of *Pseudopodadeformis* Gong & Zhong, sp. nov. Phylogenetic analyses are the same as in Cao et al. (2016) and Zhang et al. (2017). Bayesian inference strongly supported the monophyly of the *P.deformis* Gong & Zhong, sp. nov. (Fig. 5).

**Table 1.** Information on newly sequenced *Pseudopodadeformis* Gong & Zhong, sp. nov. with GenBank accession numbers.

Voucher code	Sex	COI	ITS2
HUST-SPA-22-001	♂	OQ788976	OQ797662
HUST-SPA-22-002	♀	OQ788977	OQ797663
HUST-SPA-22-003	♀	OQ788978	OQ797664
HUST-SPA-22-004	♀	OQ788979	OQ797665

## ﻿Taxonomy

**Family Sparassidae Bertkau, 1872**

**Subfamily Heteropodinae Thorell, 1873**

### ﻿Genus Pseudopoda

Taxon classificationAnimaliaAraneaeSparassidae

Jäger, 2000

255B1040-B6B7-5EAF-B8DE-893035239366

#### Type species.

*Sarotespromptus* O. Pickard-Cambridge, 1885.

#### Diagnosis (updated).

**Figure 1.**
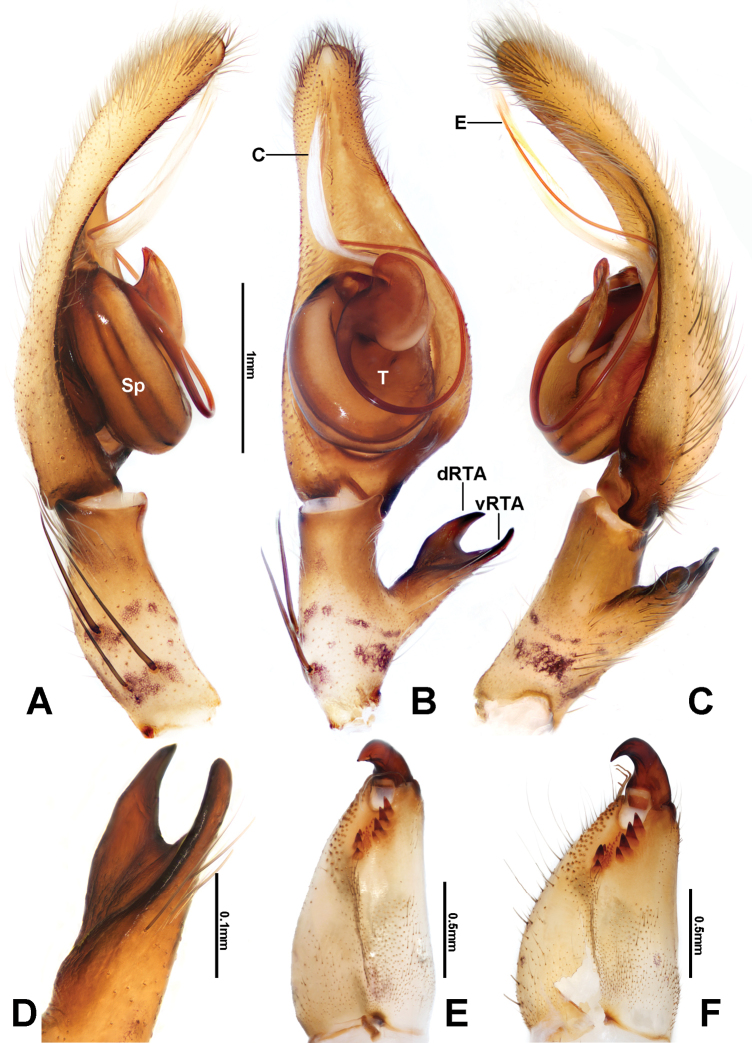
*Pseudopodadeformis* Gong & Zhong, sp. nov., male holotype (HUST-SPA-22-001), left palp (**A–C**), left male palpal tibia (**D**), and cheliceral dentition (**E, F**). **A** prolateral view **B** ventral view **C, D** retrolateral view **E** male, ventral view **F** female, ventral view. Abbreviations: C = conductor; dRTA = dorsal branch of RTA; vRTA = ventral branch of RTA; E = embolus; Sp = spermophore; T = tegulum. Scale bars: 1 mm (**A–C**); 0.1 mm (**D**); 0.5 mm (**E, F**).

**Figure 2.**
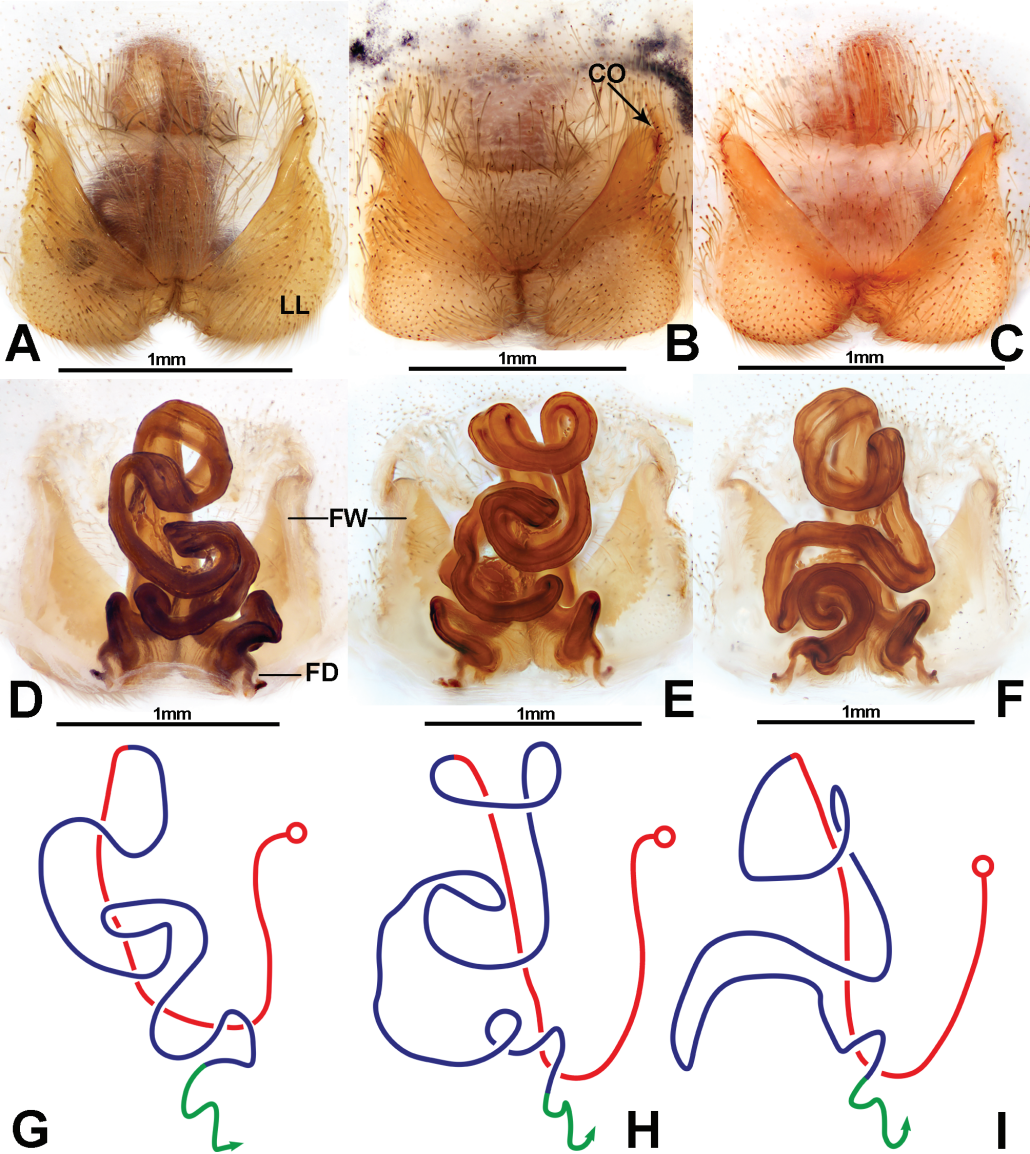
*Pseudopodadeformis* Gong & Zhong, sp. nov., female paratype (**A, D**HUST-SPA-22-002; **B, E**HUST-SPA-22-003; **C, F**HUST-SPA-22-004), epigyne (**A–C**), vulva (**D–F**), and schematic course of internal duct system (**G–I**). **A–C** ventral view **D–F** dorsal view. Abbreviations: CO = copulatory opening; FD = fertilisation duct; FW = first winding; LL = lateral lobes. Scale bars: 1 mm (**A–F**).

*Pseudopoda* was defined by Jäger (2000) according to the following combination of characters: male palp (Fig. 1A–C) with membranous conductor or absent, embolus arising on the left side of the tegulum and generally curved, RTA arising from tibia, basally or mesially and furcate or not; epigyne (Fig. 2A–C) with lateral lobes extending beyond epigastric furrow, and generally covering median septum (modified from Jäger 2000; Zhang et al. 2013; Jiang et al. 2018).

#### Distribution.

Bhutan, China, Nepal, India, Japan, Laos, Myanmar, Pakistan, Thailand and Vietnam.

### ﻿ Pseudopoda deformis

Taxon classificationAnimaliaAraneaeSparassidae

Gong & Zhong sp. nov.

**Figure 3.**
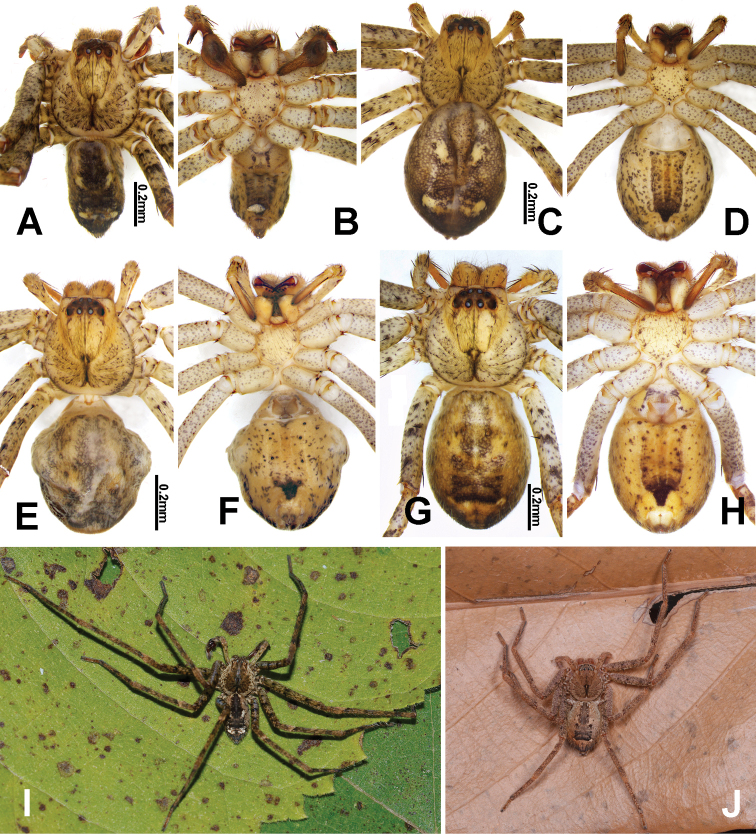
*Pseudopodadeformis* Gong & Zhong, sp. nov., habitus (**A–H**), and live specimens (**I, J**) **A, I** (HUST-SPA-22-001), holotype male, dorsal view **B** (HUST-SPA-22-001), holotype male, ventral view **C, J** (HUST-SPA-22-002), paratype female, dorsal view **D** (HUST-SPA-22-002), paratype female, ventral view **E** (HUST-SPA-22-003), paratype female, dorsal view **F** (HUST-SPA-22-003), paratype female, ventral view **G** (HUST-SPA-22-004), paratype female, dorsal view, **H** (HUST-SPA-22-004), paratype female, ventral view. Scale bars: 0.2 mm (**A–H**).

**Figure 4.**
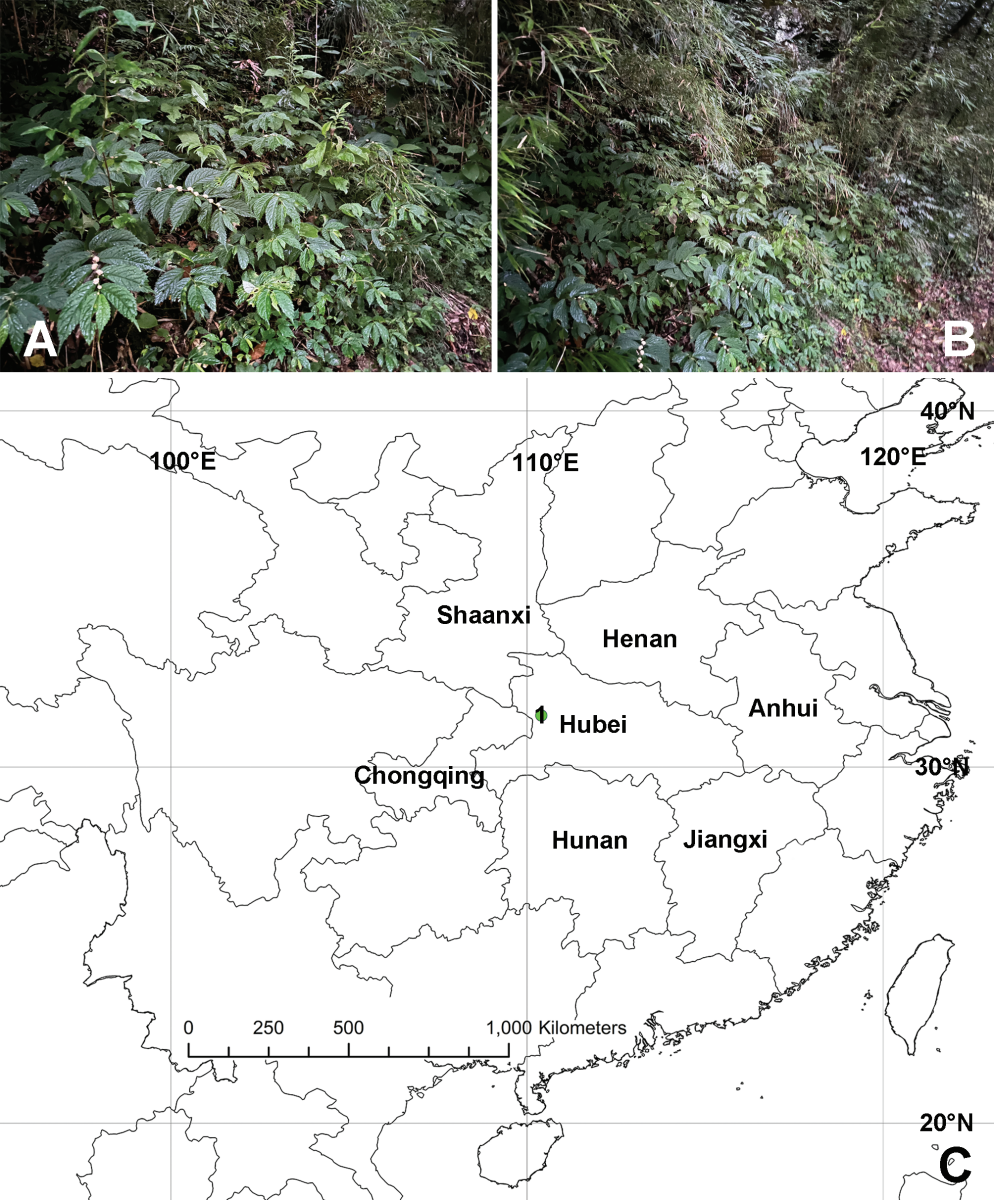
Photograph of the habitat (**A, B**) and collection locality of *Pseudopodadeformis* Gong & Zhong, sp. nov. (**C**).

**Figure 5.**
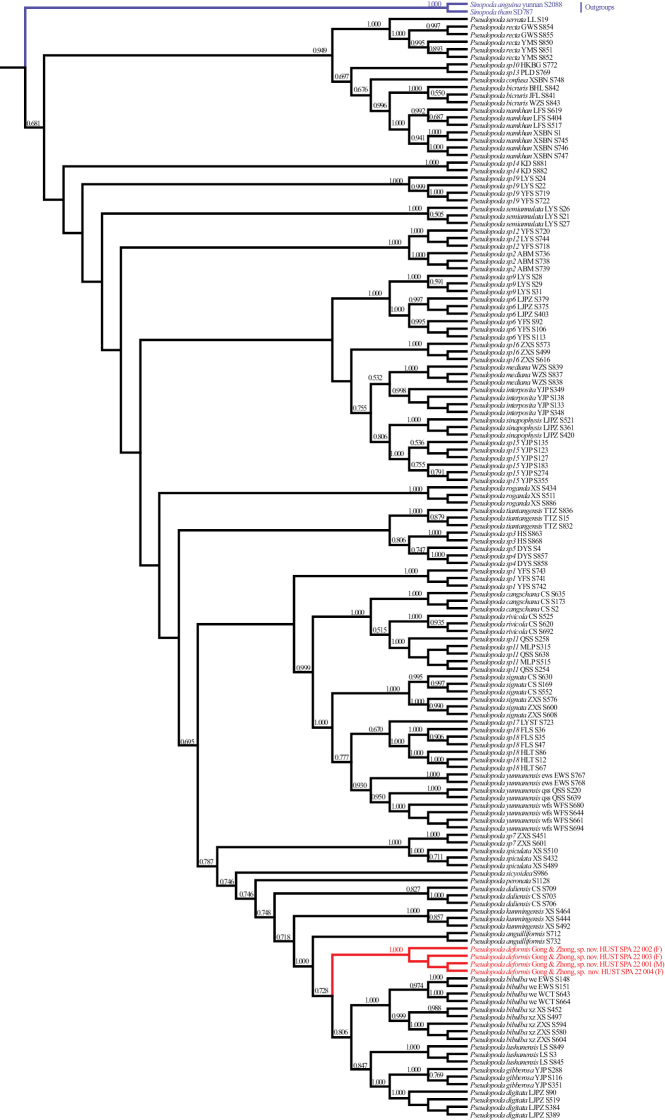
Bayesian tree based on the COI + ITS2 dataset including 146 *Pseudopoda* individuals belonging to 45 species. Numbers on nodes are posterior probabilities. Red clade indicates *Pseudopodadeformis* Gong & Zhong, sp. nov., blue clade indicates the outgroups.

6A293988-706D-51CB-9E69-0746D9AB71DD

https://zoobank.org/1F62E2C1-3556-4B86-AE30-D907B4F204CB

(变形拟遁蛛) Figs 1 , 2 , 3 , 4 , 5

#### Type material.

***Holotype* #m: China**: Hubei Province: Shennongjia Forestry District, Muyu Town, Guanmenshan Scenic Area (31.45°N, 110.40°E, 1200 m a.s.l.), 10.XII.2021, leg. Y. Zhong (HUST-SPA-22-001). ***Paratypes*: China**: Hubei Province: Same locality, 1#f (HUST-SPA-22-002), 1#f (HUST-SPA-22-003), 1#f (HUST-SPA-22-004), 3#m, 4#f.

#### Etymology.

The specific name is derived from the Latin word *deformis*, -*a*, -*um*, meaning distorted, referring to the shape of the internal ducts of the female vulva.

#### Diagnosis.

Males of *Pseudopodadeformis* Gong & Zhong, sp. nov. are similar to those of *P.jiangi* Zhang, Jäger & Liu, 2023 (Zhang et al. 2023: figs 130, 131), *P.lushanensis* (Wang, 1990) (Quan et al. 2014: figs 4A–C, 5A–C) and *P.shuqiangi* Jäger & Vedel, 2007 (Jäger and Vedel 2007: figs 73–75) in having a long, filiform embolus. They can be distinguished from the two congeners by the following combination of characters: (1) Embolus arising from tegulum at 1:00-o’clock position, then curving downward (8:30-o’clock position, upward in *P.lushanensis* and *P.shuqiangi*); (2) The basal part of embolus is oval (circular in *P.jiangi*); (3) The tip of the conductor is straight and extends to approximately the tip of the cymbium in ventral view (not in *P.lushanensis* and *P.shuqiangi*); and (4) RTA arising medially from tibia (subdistally in *P.lushanensis*; basally in *P.shuqiangi*) (Fig. 1A–C). The females of this species can be separated from other *Pseudopoda* species by their unique internal ducts of the vulva, which are curved longitudinally, forming a narrow triangle or trapezoidal shape (Fig. 2D–I).

#### Description.

**Male.**PL 4.9, PW 3.4, AW 2.3, OL 4.6, OW 3.4. Eyes: AME 0.24, ALE 0.26, PME 0.27, PLE 0.33, AME–AME 0.21, AME–ALE 0.12, PME–PME 0.23, PME–PLE 0.16, AME–PME 0.29, ALE–PLE 0.18, CHAME 0.27, CHALE 0.33. Setation: Palp: 131, 101, 2101; Fe: I–III 323, IV 321; Pa: I–IV 101; Ti: I–II 2228, III–IV 2126; Mt: I–II 2024, III 2026, IV 3036. Measurements of palp and legs: Palp 7.4 (2.1, 0.9, 1.3, –, 3.1), I 27.0 (7.1, 1.5, 8.2, 7.8, 2.4), II 29.6 (7.8, 1.5, 8.8, 8.9, 2.6), III 21.6 (6.0, 1.3, 6.1, 6.3, 1.9), IV 24.5 (6.5, 1.3, 6.9, 7.6, 2.2). Leg formula: II-I-IV-III. Chelicerae with three promarginal and four retromarginal teeth, and with ~51 denticles (Fig. 1E). Carapace yellowish brown dorsally, margin with black patches, with shallow fovea and radial furrows. Chelicerae deep reddish brown. Sternum yellow with lots of random black spots. Endites and labium pale yellowish brown. Legs brown, with dark dots randomly distributed and covered by short spines and seta. Opisthosoma black-brown dorsally, without spots. Opisthosoma uniformly yellowish brown with some black patches ventrally (Fig. 3A, B).

Cymbium approximately 2 times longer than tibia (Fig. 1A–C). The basal part of conductor is obscured in ventral view (Fig. 1B) by embolus base. Basal part of conductor slightly sclerotized (Fig. 1C). Embolus slender, encircling the tegulum counter-clockwise, ventrally pointed (Fig. 1B). RTA distally bifurcate, pincer-shaped in ventral view, dRTA moderately pointed at tip (Fig. 1B, D).

**Female.**PL 5.0, PW 4.8, AW 2.8, OL 6.7, OW 4.7. Eyes: AME 0.25, ALE 0.34, PME 0.26, PLE 0.38, AME–AME 0.18, AME–ALE 0.08, PME–PME 0.24, PME–PLE 0.18, AME–PME 0.29, ALE–PLE 0.17, CHAME 0.35, CHALE 0.45. Setation: Palp: 131, 101, 2121, 1014; Fe: I–III 323, IV 321; Pa: I–IV 000; Ti: I–II 222(10), III–IV 2126; Mt: I–II 2024, III–IV 2026. Measurements of palp and legs: Palp 6.4 (2.1, 0.7, 1.0, –, 2.6), I 19.3 (5.5, 1.3, 5.6, 5.1, 1.8), II 20.5 (5.9, 1.3, 6.1, 5.4, 1.8), III 15.4 (4.6, 1.1, 4.5, 3.7, 1.5), IV 17.0 (5.0, 1.1, 4.5, 4.6, 1.8). Leg formula: II-I-IV-III. Chelicerae with three promarginal and four retromarginal teeth, and with ~46 denticles (Fig. 1F).

Epigynal field almost as wide as long, the anterior margins of lateral lobes forming a V-shape, median margin of lateral lobes united, internal duct systems not visible through cuticle, fertilisation ducts arising postero-laterally. In the dorsal view, internal duct systems differ extremely, and there is no regularity in the direction and structure of internal pipeline (Fig. 2).

Colouration as in males, opisthosoma brown dorsally (Fig. 3C–H).

#### Habitat.

The specimens were collected on leaves at night with bare hands (Fig. 4A, B).

#### Distribution.

Known only from Hubei Province, China (Fig. 4C).

#### Remarks.

The monophyly of *Pseudopodadeformis* Gong & Zhong, sp. nov. is highly supported by molecular phylogenetic results based mainly on Chinese *Pseudopoda* species (Fig. 5).

## ﻿Discussion

We examined all specimens from Shennongjia (4 males, 7 females) and found no variation in the male palp. However, the females had different internal ducts in their vulva, which is not known to occur in other *Pseudopoda* spiders. In this paper, matching of the sexes of *Pseudopodadeformis* Gong & Zhong, sp. nov. was done using morphological and molecular data (Figs 3, 5). Females of this species resemble most *Pseudopoda* species having the median margin of the lateral lobes converged medially with the anterior part V-shaped, but they can be distinguished from these species by the longitudinally bent internal duct system of the vulva (Fig. 2A–F). As shown in Fig. 2G–I, the female vulva is divided into three parts schematically to show the course of the internal duct system, leading to an interesting discovery. The first part is represented by a red line and is U-shaped in all three females examined. The third part is represented by a green line and is an inverted S-shaped. Variability occurs in the second part which is shown by the blue line, and there are irregularities to this variation.

## Supplementary Material

XML Treatment for Pseudopoda

XML Treatment for Pseudopoda deformis

## References

[B1] Bertkau P (1872) Über die Respirationsorgane der Araneen. Archiv für Naturgeschichte 38: 208–233.

[B2] Cao XW Liu J Chen J Zheng G Kuntner M Agnarsson I (2016) Rapid dissemination of taxonomic discoveries based on DNA barcoding and morphology. Scientific Reports 6(1): 37066. 10.1038/srep37066 27991489 PMC5171852

[B3] Folmer M Black W Lutz R Vrijenhoek R (1994) DNA primers for amplification of mitochondrial cytochrome c oxidase subunit I from diverse metazoan invertebrates. Molecular Marine Biology and Biotechnology 3: 294–299. 7881515

[B4] Gong LJ Zhong Y (2020) Redescription of *Pseudopodataibaischana* (Araneae, Sparassidae), with the first description of the female. ZooKeys 991: 111–119. 10.3897/zookeys.991.56969 33223901 PMC7674379

[B5] Jäger P (2000) Two new heteropodine genera from southern continental Asia (Araneae: Sparassidae). Acta Arachnologica 49(1): 61–71. 10.2476/asjaa.49.61

[B6] Jäger P Vedel V (2007) Sparassidae of China 4. The genus *Pseudopoda* (Araneae: Sparassidae) in Yunnan Province. Zootaxa 1623(1): 1–38. 10.11646/zootaxa.1623.1.1

[B7] Jiang TY Zhao QY Li SQ (2018) Sixteen new species of the genus *Pseudopoda* Jäger, 2000 from China, Myanmar, and Thailand (Sparassidae, Heteropodinae). ZooKeys 791: 107–161. 10.3897/zookeys.791.28137 PMC6205991 30386156

[B8] Quan D Zhong Y Liu J (2014) Four *Pseudopoda* species (Araneae: Sparassidae) from southern China. Zootaxa 3754(5): 555–571. 10.11646/zootaxa.3754.5.2 24869707

[B9] Thorell T (1873) Remarks on synonyms of European spiders. Part IV. C. J. Lundström, Uppsala, 375–645.

[B10] WSC (2023) World Spider Catalog, version 24. Natural History Museum, Bern. 10.24436/2 [Accessed 10.04.2023]

[B11] Zhang F Zhang BS Zhang ZS (2013) New species of *Pseudopoda* Jäger, 2000 from southern China (Araneae, Sparassidae). ZooKeys 361: 37–60. 10.3897/zookeys.361.6089 PMC3867119 24363596

[B12] Zhang H Jäger P Liu J (2017) One new *Pseudopoda* species group (Araneae: Sparassidae) from Yunnan Province, China, with description of three new species. Zootaxa 4318(2): 271–294. 10.11646/zootaxa.4318.2.3

[B13] Zhang H Zhong Y Zhu Y Agnarsson I Liu J (2021) A molecular phylogeny of the Chinese *Sinopoda* spiders (Sparassidae, Heteropodinae): implications for taxonomy. PeerJ 9: e11775. [26 pp] 10.7717/peerj.11775 PMC8381878 34484980

[B14] Zhang H Zhu Y Zhong Y Jäger P Liu J (2023) A taxonomic revision of the spider genus *Pseudopoda* Jäger, 2000 (Araneae: Sparassidae) from East, South and Southeast Asia. Megataxa 9(1): 1–304. 10.11646/megataxa.9.1.1

